# The inclusion of e-cigarettes and heated tobacco products in smoke-free home and car rules: A cross-sectional survey of adults in Armenia and Georgia

**DOI:** 10.18332/tid/189200

**Published:** 2024-06-04

**Authors:** Varduhi Hayrumyan, Zhanna Sargsyan, Arevik Torosyan, Ana Dekanosidze, Lilit Grigoryan, Nour Alayan, Michelle C. Kegler, Lela Sturua, Varduhi Petrosyan, Alexander Bazarchyan, Regine Haardörfer, Yuxian Cui, Carla J. Berg

**Affiliations:** 1 Turpanjian College of Health Sciences, American University of Armenia, Yerevan, Republic of Armenia; 2 National Institute of Health named after academician S. Avdalbekyan, Ministry of Health, Yerevan, Republic of Armenia; 3 Georgia National Center for Disease Control and Public Health, Tbilisi, Georgia; 4 Tbilisi State Medical University, Tbilisi, Georgia; 5 Department of Behavioral, Social, and Health Education Sciences, Rollins School of Public Health, Emory University, Atlanta, United States; 6 Petre Shotadze Tbilisi Medical Academy, Tbilisi, Georgia; 7 Department of Prevention and Community Health, Milken Institute School of Public Health, The George Washington University; 8 George Washington Cancer Center, George Washington University, Washington, United States

**Keywords:** smoke-free homes and cars, alternative tobacco products, e-cigarettes, heated tobacco products, secondhand exposure

## Abstract

**INTRODUCTION:**

Understanding who includes e-cigarettes and heated tobacco products (HTPs) in smoke-free home or car rules could inform public health interventions, particularly in countries with high smoking prevalence and recently implemented national smoke-free laws, like Armenia and Georgia.

**METHODS:**

In 2022, we conducted a cross-sectional survey among 1468 adults in 28 Armenian and Georgian communities (mean age=42.92 years; 51.4% female, 31.6% past-month smoking). Multilevel regression (accounting for clustering within communities; adjusted for sociodemographics and cigarette use) examined e-cigarette/HTP perceptions (risk, social acceptability) and use intentions in relation to: 1) including e-cigarettes/HTPs in home and car rules among participants with home and car rules, respectively (logistic regressions); and 2) intention to include e-cigarettes/HTPs in home rules (linear regression, 1 = ‘not at all’ to 7 = ‘extremely’) among those without home rules.

**RESULTS:**

Overall, 72.9% (n=1070) had home rules, 86.5% of whom included e-cigarettes/HTPs; 33.9% (n=498) had car rules, 81.3% of whom included e-cigarettes/HTPs. Greater perceived e-cigarette/HTP risk was associated with including e-cigarettes/HTPs in home rules (AOR=1.28; 95% CI: 1.08–1.50) and car rules (AOR=1.46; 95% CI: 1.14–1.87) and next-year intentions to include e-cigarettes/HTPs in home rules (β=0.38; 95% CI: 0.25–0.50). Lower e-cigarette/HTP use intentions were associated with including e-cigarettes/HTPs in home rules (AOR=0.75; 95% CI: 0.63–0.88). While perceived social acceptability was unassociated with the outcomes, other social influences were: having children and no other household smokers was associated with including e-cigarettes/HTPs in car rules, and having children was associated with intent to include e-cigarettes/HTPs in home rules.

**CONCLUSIONS:**

Interventions to address gaps in home and car rules might target e-cigarette/HTP risk perceptions.

## INTRODUCTION

Comprehensive smoke-free policies, as recommended by the World Health Organization (WHO) Framework Convention on Tobacco Control (FCTC), have been instrumental in reducing secondhand smoke exposure (SHSe) in various public spaces, leading to improved health outcomes^1^. However, the persistence of SHSe in private settings, such as homes and vehicles, underscores the need to implement effectively smoke-free rules in these personal environments^2^. Despite the absence of direct guidelines in the WHO FCTC regarding smoke-free regulations in private settings, research suggests that public smoke-free restrictions lead to the adoption of voluntary implementation of restrictions in private settings such as homes and cars^3^. Voluntary adoption of these rules can effectively reduce smoking rates, increase smoking cessation efforts, and discourage smoking initiation^2^.

The increasing prevalence of newer tobacco products, such as e-cigarettes and heated tobacco products (HTPs), has added complexity to the issue of SHSe^4^. While traditional cigarettes remain the primary form of tobacco consumption globally, the utilization of these alternative tobacco products is steadily growing, especially in European countries^5,6^. In contrast to the well-established evidence regarding the adverse health effects of SHSe from conventional cigarettes, the potential risks associated with e-cigarette and HTP byproduct exposure are less well understood, but may contain various harmful chemicals (e.g. nicotine, carcinogens) with potentially negative implications for those who use them and for bystanders^7,8^. Of particular concern is the exposure in private indoor environments^8^.

Following the guidelines outlined by the WHO FCTC, regulations for these newer tobacco products should ideally mirror those for conventional tobacco products, prohibiting them in all indoor areas or, at the very least, in spaces where smoking is already banned^9^. While at least 74 countries (representing over one-fourth of the global population) have comprehensive smoke-free air laws^7^, a 2022 review of over 130 countries found that 66 (about half) restricted or banned e-cigarette use in public places, with only 23 referencing HTPs^10^. However, recent studies have indicated a concerning trend of frequent e-cigarette and HTP use in settings where smoking is prohibited, including workplaces and restaurants^11^. Moreover, the evasion of smoke-free regulations has been identified as one of the motivators for using e-cigarettes^12^ and HTPs^13^, underscoring the need to address these increasingly prevalent products. Furthermore, e-cigarette and HTP byproduct exposure in private settings is problematic in the absence of formal regulations for private settings^8^, which are seldom in place in most countries^14^ and are mainly voluntary^15^. E-cigarette or HTP use in private settings is associated with a perceived lower risk of their byproducts than SHS, particularly in homes without smoke-free rules or restrictions^16^. However, voluntary restrictions on e-cigarette use at home may reduce byproduct exposure^17^.

Addressing e-cigarette and HTP use in private settings is particularly crucial in regions with high smoking prevalence, like many low- and middle-income countries. Armenia and Georgia (both middle-income countries) have high male tobacco use rates (56.1% and 49.5%, respectively) but lower rates among women (2.6% and 8.5%, respectively)^18,19^, as well as high rates of SHSe (past-month: 74.2%; daily: 24.4%)^20,21^. Armenia and Georgia ratified the WHO FCTC in 2004 and 2006, respectively, and both countries have shown substantial progress in adopting progressive tobacco control legislation (Georgia in 2017–2018 and Armenia in 2020) and enforcing public smoke-free laws, taking full effect in Georgia in 2018 and Armenia in 2022. However, a substantial percentage of households in these countries still allow smoking: >75% of households in Armenia and about 50% of households in Georgia^21^. Adding to the complexities and challenges of the tobacco use context in these countries is the emergence of e-cigarettes and HTPs in their markets. Although national estimates of e-cigarette and HTP use in these countries are sparse, available estimates indicate adult past-month use of e-cigarettes and HTPs of about 3%, respectively, in Armenia^18^ and about 1.5%, respectively, in Georgia^22^.

Given the persistent issue of SHS and the increasing prevalence of e-cigarette and HTP use, it is crucial to assess the inclusion of these products within existing smoke-free rules in private settings. Understanding factors associated with private smoke-free rules encompassing e-cigarettes and HTPs, is particularly imperative given the absence of research addressing this issue and the potential to identify related intervention targets. Smoke-free home interventions have largely integrated Social Cognitive Theory^23^, emphasizing the particular role of social factors, like social norms, or cognitive factors, such as perceived risk. Thus, this study examined e-cigarette/HTP use intentions and perceptions (risk, social acceptability), as well as sociodemographic and tobacco use-related factors, in relation to including e-cigarettes and HTPs in smoke-free home and car rules, and the likelihood of implementing home rules, including e-cigarettes and HTPs among Armenian and Georgian adults.

## METHODS

### Study overview

The current study analyzed cross-sectional survey data collected in 2022 among 1468 adults in 28 Armenian and Georgian communities (i.e. municipalities). These data were from a larger study examining the effectiveness of local coalitions in promoting smoke-free policies and reducing SHSe, which entailed a matched-pairs community randomized controlled trial that was launched in the Fall of 2018 and culminated in 2022 (detailed methods and results presented elsewhere)^24^. The Institutional Review Boards of Emory University (#IRB00097093), the National Academy of Sciences of the Republic of Armenia (#IRB00004079), American University of Armenia (#AUA-2017-013), and National Center for Disease Control and Public Health of Georgia (#IRB00002150) approved this study.

### Data collection

In each of the 28 communities (intervention and control), we conducted population-level surveys at baseline in 2018 (October–November) and at follow-up in 2022 (May–June). The analyses of the current study focused only on the 2022 survey data. In both countries, we acquired census data for households within the municipal boundaries. Sampling strategies varied across countries, as household data were available in Armenia but not in Georgia; in Georgia, we used ‘clusters’ (i.e. geographically defined areas of 150 households). In both countries, we acquired census data for households within the municipal boundaries. To identify target participants (i.e. aged 18–64 years) in each household, we employed the KISH method (a systematic sampling technique used to randomly select household survey respondents with equal probability)^25^, aiming for 50 participants per community (the sample size was based on power calculations for the parent study^24^, but allows for the detection of small to medium effects in the current analyses).

In Armenia, households in each city were ordered using a random number generator. We began assessments from the beginning of the list and continued until recruitment targets were met. In 2022, 1140 households were visited, with 890 (78.1%) deemed eligible, 763 (85.7%) of which participated. In Georgia, we identified 5 clusters per city for the sampling, and then we used the random walking method to select 15 households per cluster^26^. In 2022, 916 households were visited, with 839 (91.6%) deemed eligible, 705 (84.0%) of which participated. Participants provided verbal informed consent before participating.

### Measures

The questionnaire was originally developed in English, translated into Armenian and Georgian languages, and then back-translated. Variables included in analyses were based on the literature, as certain demographics (i.e. age, sex, education level)^27^, household composition (i.e. children or other smokers in the home)^27^, national tobacco control context (i.e. Armenia vs Georgia)^1^, and use perceptions and intentions have been associated with smoke-free rules in personal settings^23^. The inclusion of use perceptions and intentions is also based on health behavior theories (e.g. Social Cognitive Theory)^23^.

*Outcomes*

We assessed participants’ smoke-free home and car rules to determine subsamples for each analysis. Smoke-free home rules were assessed by asking: ‘Which of the following statements best describes the smoking rules in your home: smoking in your home is allowed, smoking in your home is generally not allowed with certain exceptions, smoking in your home is never allowed, or there are no rules about smoking in your home?’. Response options were: a) Allowed; b) Not allowed but with exceptions; c) Never allowed; and d) No rules (any rules = b or c; full rules = c; partial rules = b)^28,29^. Smoke-free vehicle rules were assessed by asking: ‘Which statement best describes the rules about smoking in your household vehicles (cars or trucks)?’. Response options were: a) Allowed in all vehicles; b) Smoking is sometimes allowed in some vehicles; c) Smoking is never allowed in any vehicle; d) There are no rules about smoking in the vehicles; and e) We don’t own a vehicle. Among those with vehicles: any rules = b or c; full rules = c; partial rules = b)^28,29^.

We then assessed our outcomes. To assess whether these rules included e-cigarettes and HTPs, participants reporting ‘never allowed’ or ‘not allowed but with exceptions’ for homes and cars, respectively, were asked: ‘Does this rule also ban the use of (check all that apply): e-cigarettes, heated tobacco products like IQOS?’. We operationalized the inclusion of e-cigarettes/HTPs in the home or car rules, indicating that the rules covered both e-cigarettes and HTPs (Table 1 footnote). Participants were also asked: ‘In the next year, how likely are you to implement a rule in your home banning – or continuing to ban – the indoor use of e-cigarettes, heated tobacco products like IQOS?’ (1 = ‘not at all’ to 7 = ‘extremely likely’). Scores from these two items were averaged to create an index score for intention to establish home rules, including e-cigarettes/HTPs.

**Table 1:** Bivariate analyses examining correlates of having home and car rules (full or partial) that include e-cigarettes and HTPs among those with full or partial home or car rules, respectively, and intention to include e-cigarettes and HTPs in home rules in the next year among those without rules including e-cigarettes and HTPs, cross-sectional survey of adults in Armenia and Georgia, 2022 (N=1468)

	*Home rules include e-cigarettes and HTPs ^a^*				*Car rules include e-cigarettes and HTPs ^b^*				*Intention to include e-cigarettes and HTPs in home rules ^c^ (N=540)*	
	*Total*	*No*	*Yes*		*Total*	*No*	*Yes*			
	*N=1070 (100.0)*	*N=144 (13.5)*	*N=926 (86.5)*		*N=498 (100.0)*	*N=93 (18.7)*	*N=405 (81.3)*			
	*n (%)*	*n (%)*	*n (%)*	*p*	*n (%)*	*n (%)*	*n (%)*	*p*	*M (SD) or r*	*p*
**Sociodemographics**										
**Country**										
Armenia	469 (43.8)	96 (66.7)	373 (40.3)	**<0.001**	244 (49.0)	59 (63.4)	185 (45.7)	**0.002**	3.82 (2.26)	**<0.001**
Georgia	601 (56.2)	48 (33.3)	553 (59.7)		254 (51.0)	34 (36.6)	220 (54.3)		2.72 (2.25)	
**Age** (years), mean (SD)	42.89 (13.81)	44.13 (14.36)	42.70 (13.73)	0.250	41.93 (13.22)	41.45 (13.37)	42.04 (13.20)	0.697	-0.005	0.911
**Gender**										
Male	490 (45.8)	70 (48.6)	420 (45.4)	0.466	247 (49.6)	45 (48.4)	202 (49.9)	0.796	3.18 (2.44)	**0.001**
Female	580 (54.2)	74 (51.4)	506 (54.6)		251 (50.4)	48 (51.6)	203 (50.1)		3.91 (2.66)	
**Education level**										
≤High school	286 (26.7)	33 (22.9)	253 (27.3)	0.266	110 (22.1)	17 (18.3)	93 (23.0)	0.326	2.97 (2.48)	**0.003**
>High school	784 (73.3)	111 (77.1)	673 (72.7)		388 (77.9)	76 (81.7)	312 (77.0)		3.71 (2.57)	
**Children aged <18 years in the home**										
Yes	537 (50.2)	68 (47.2)	469 (50.6)	0.444	282 (56.6)	39 (41.9)	243 (60.0)	**0.002**	3.87 (2.50)	**0.003**
No	533 (49.8)	76 (52.8)	457 (49.4)		216 (43.4)	54 (58.1)	162 (40.0)		3.21 (2.59)	
**Other smokers in the home**										
Yes	388 (36.3)	52 (36.1)	336 (36.3)	0.968	180 (36.1)	43 (46.2)	137 (33.8)	**0.025**	3.64 (2.56)	0.303
No	682 (63.7)	92 (63.9)	590 (63.7)		318 (63.9)	50 (53.8)	268 (66.2)		3.41 (2.57)	
**Past-month cigarette use**										
Yes	279 (26.1)	51 (35.4)	228 (24.6)	**0.006**	118 (23.7)	29 (31.2)	89 (22.0)	0.060	2.90 (2.36)	**<0.001**
No	791 (73.9)	93 (64.6)	698 (75.4)		380 (76.3)	64 (68.8)	316 (78.0)		4.00 (2.62)	
**Home or car rules**										
No	d0 (0.0)	0 (0.0)	0 (0.0)	**<0.001**	0 (0.0)^e^	0 (0.0)	0 (0.0)	**<0.001**	3.58 (2.58)^d^	0.076
Partial	283 (26.4)	85 (59.0)	198 (21.4)		119 (23.9)	41 (44.1)	78 (19.3)		2.98 (2.39)	
Full	787 (73.6)	59 (41.0)	728 (78.6)		379 (76.1)	52 (55.9)	327 (80.7)		3.88 (2.62)	
**E-cigarette/HTP use intention/perceptions, mean** (SD)^c^										
Use intentions (of e-cigarettes/HTPs)	1.37 (1.16)	1.83 (1.78)	1.29 (1.01)	**<0.001**	1.35 (1.16)	1.48 (1.33)	1.31 (1.12)	0.223	-0.101	**0.019**
Perceived risk	6.02 (1.40)	5.39 (1.67)	6.11 (1.33)	**<0.001**	6.06 (1.35)	5.74 (1.49)	6.14 (1.31)	**0.012**	0.335	**<0.001**
Perceived social acceptability	2.30 (1.53)	2.00 (1.36)	2.35 (1.55)	**0.011**	2.26 (1.49)	2.13 (1.60)	2.29 (1.47)	0.346	0.005	0.053

HTPs: heated tobacco products. r: Pearson’s correlation coefficient. Statistical significance set at p<0.05 (two-tailed tests).

a 98.3% of those with e-cigarette rules apply them to HTPs, and vice versa.

b 1 missing. 97.4% of those with car rules for e-cigarettes apply them to HTPs. 98.1% of those with car rules for HTPs apply them to e-cigarettes.

c Correlations between e-cigarette and HTP items significant (p<0.001) for likelihood to ban in home: r=0.089; use intentions: r=0.83; and perceived harm; r=0.89, addictiveness: r=0.92, and social acceptability: r=0.85. For all 3 index measures, Cronbach’s alpha=0.91.

d Home rules.

e Car rules.

*Theory-informed factors of interest*

E-cigarette and HTP use intentions were assessed by asking: ‘How likely are you to try or continue to use e-cigarettes, heated tobacco products such as IQOS, in the next year?’ (1 = ‘not at all’ to 7 = ‘extremely’)^28,29^. Scores from these two items were averaged to create an e-cigarette/HTP use intention index score.

Perceptions of e-cigarette and HTP harms, addictiveness, and social acceptability were assessed by asking: ‘How harmful to your health, addictive, socially acceptable among your peers, do you think the e-cigarettes, heated tobacco products such as IQOS are?’ (1 = ‘not at all’ to 7 = ‘extremely’)^28,29^. Scores from the four items assessing perceived harm and addictiveness of e-cigarettes and HTPs were averaged to create an e-cigarette/HTP risk perception index score. Scores from the two items assessing the social acceptability of e-cigarettes and HTPs were averaged to create an e-cigarette/HTP social acceptability index score.

*Covariates: sociodemographic and tobacco use characteristics*

Current analyses included the following: country, age, sex, education level, having children in the home, other smokers in the home, and past 30-day cigarette, e-cigarette, and HTP use.

### Data analysis

Descriptive analyses were conducted to characterize the sample and to examine data (e.g. e-cigarette/HTP use intentions and perceptions, intention to include e-cigarettes/HTPs in home roles) for normality of distribution. Next, bivariate analyses were used to examine associations between factors of interest and covariates in relation to each outcome (for categorical outcomes, using chi-squared tests for categorical variables and t-tests and one-way ANOVAs for continuous variables; for the continuous outcome of intention, using t-tests and one-way ANOVAs for categorical variables and Pearson’s r for continuous variables).

Next, to examine factors associated with our three outcomes, we conducted multilevel multivariable regression models using random effects (to account for clustering within communities) adjusted for covariates (sociodemographics, current cigarette use). Binary logistic regression models were conducted to include e-cigarettes/HTPs in home and car rules, respectively. Also, they included the level of home and car rules in place (i.e. no, partial, full). Linear regression was conducted with the intention to include e-cigarettes/HTPs in home rules. Analyses were conducted in SPSS v.27, and alpha was set at 0.05 (for two-tailed tests ).

## RESULTS

### Participant characteristics

In the overall sample (n=1468), the majority were from Armenia (52.0%), female (51.4%), and high school educated or more (73.1%); less than half had children aged <18 years (49.4%) or other smokers in the home (39.9%). Past-month cigarette, e-cigarette, and HTP use were 31.6%, 3.2%, and 2.7%, respectively. Average index scores (1 = ‘not at all’ to 7 = ‘extremely’) for next-year e-cigarette/HTP use intentions, perceived risk, and social acceptability were 1.47 (SD=1.33), 5.85 (SD=1.55), and 2.32 (SD=1.54), respectively.

### Inclusion of e-cigarettes and HTPs in smoke-free home rules

Of the 72.9% (n=1070) participants with smoke-free home rules, 86.5% included e-cigarettes/HTPs in those rules. Bivariate analyses showed (Table 1) that including e-cigarettes/HTPs in smoke-free home rules was associated with being from Georgia (p<0.001), no past-month smoking (p<0.006), having full (vs partial) smoke-free home rules (p<0.001), having lower e-cigarette/HTP use intentions (p<0.001), greater perceived e-cigarette/HTP risk (p<0.001), and greater perceived e-cigarette/HTP social acceptability (p=0.011).

**Figure 1:**
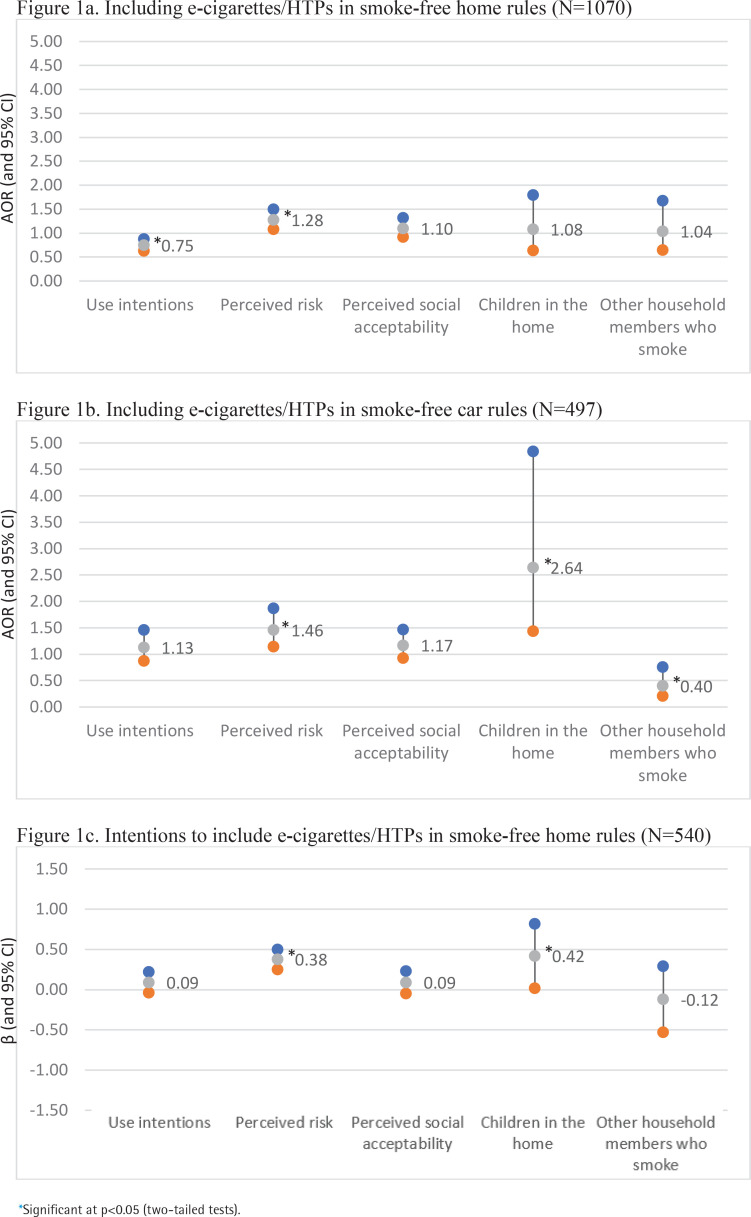
Adjusted odds ratios (AORs) or coefficients (β) and 95% confidence intervals (CIs) from multilevel regression models assessing theory-relevant factors in relation to including e-cigarettes and heated tobacco products (HTPs) in smoke-free home and car rules

Adjusted multilevel binary logistic regression analysis (Table 2) revealed that factors associated with including e-cigarettes/HTPs in home rules were being from Georgia (AOR=4.14; 95% CI: 1.15–14.96, p=0.030), having full (vs partial) smoke-free home rules (AOR=6.42; 95% CI: 3.94–10.74, p<0.001), lower e-cigarette/HTP use intentions (AOR=0.75; 95% CI: 0.63–0.88, p<0.001), and greater perceived e-cigarette/HTP risk (AOR=1.28; 95% CI: 1.08–1.50, p=0.004) (Selected findings are presented in Figure 1a.).

**Table 2:** Multivariable regression models examining correlates of having home and car rules (full or partial) that include e-cigarettes and HTPs among those with full or partial home or car rules, respectively, and intention to include e-cigarettes and HTPs in home rules in the next year among those without rules including e-cigarettes and HTPs, cross-sectional survey of adults in Armenia and Georgia, 2022 (N=1468)

	*Home rules include e-cigarettes and HTPs ^a^*			*Car rules include e-cigarettes and HTPs ^b^*			*Intention to include e-cigarettes and HTPs in home rules ^c^*		
	*AOR*	*95% CI*	*p*	*AOR*	*95% CI*	*p*	*β*	*95% CI*	*p*
**Sociodemographics**									
Country – Georgia (Ref: Armenia)	4.14	1.15–15.0	**0.030**	2.01	0.61–6.64	0.255	-0.64	-1.46–0.18	0.124
Age (years)	0.99	0.97–1.00	0.114	1.00	0.98–1.02	0.824	0.01	-0.01–0.02	0.268
Gender – Female (Ref: Male)	0.99	0.54–1.84	0.982	0.57	026–1.25	0.157	-0.22	-0.79–0.36	0.457
Education level – > High school (Ref: ≤ High school)	1.29	0.74–2.28	0.372	1.91	0.83–3.96	0.137	0.33	-0.14–0.80	0.169
Children aged <18 years in the home – Yes (Ref: No)	1.08	0.64–1.80	0.859	2.64	1.44–4.84	**0.002**	0.42	0.02–0.82	**0.042**
Other smokers in the home – Yes (Ref: No)	1.04	0.65–1.68	0.780	0.40	0.21–0.76	**0.005**	-0.12	-0.53–0.29	0.558
Past-month cigarette use – Yes (Ref: No)	0.87	0.45–1.68	0.669	0.64	0.27–1.55	0.324	-0.82	-1.40 – -0.23	**0.007**
**Home or car rules** (Ref: see notes)	^d^			^e^			^d^Ref		
Partial	Ref			Ref			-0.12	-0.68–0.45	0.684
Full	6.42	3.94–10.74	**<0.001**	5.95	3.03–11.68	**<0.001**	0.37	-0.29–1.04	0.272
**E-cigarette/HTP use intentions and perceptions**									
Use intentions	0.75	0.63–0.88	**<0.001**	1.13	0.87–1.46	0.378	0.09	-0.04–0.22	0.164
Perceived risk	1.28	1.08–1.50	**0.004**	1.46	1.14–1.87	**0.003**	0.38	0.25–0.50	**<0.001**
Perceived social acceptability	1.10	0.92–1.32	0.283	1.17	0.93–1.47	0.172	0.09	-0.05–0.23	0.212

AOR: adjusted odds ratio; adjusted multilevel regression models accounting for clustering within communities. β: coefficient. Statistical significance set at p<0.05 (two-tailed tests).

a Binary logistic regression (N=1070).

b Binary logistic regression (N=497).

c Linear regression (N=540).

d Home rules.

e Car rules. HTPs: heated tobacco products.

### Inclusion of e-cigarettes and HTPs in smoke-free car rules

Of the 33.9% (n=498) participants with smoke-free car rules, 81.3% included e-cigarettes/HTPs in those rules. In bivariate analysis (Table 1), the inclusion of e-cigarettes/HTPs in smoke-free car rules among those who owned vehicles and had car rules was associated with being from Georgia (p=0.002) and having children in the home (p=0.002), no other smokers in the home (p=0.025), full (vs partial) car rules (p<0.001), and greater perceived e-cigarette/HTP risk (p=0.012).

In adjusted multilevel binary logistic regression analysis (Table 2), factors associated with including e-cigarettes/HTPs in car smoke-free rules were having children (AOR=2.64; 95% CI: 1.44–4.84, p=0.002), no other smokers in the home (AOR=0.40; 95% CI: 0.21–0.76, p=0.005), full (vs partial) car rules (AOR=5.95; 95% CI: 3.03–11.68, p<0.001), and greater perceived e-cigarette/HTP risk (AOR=1.46; 95% CI: 1.14–1.87, p=0.003) (Selected findings are presented in Figure 1b.)

### Intention to include e-cigarettes and HTPs in home rules

In the bivariate analysis (Table 1), in those without home rules, including e-cigarettes/HTPs (n=540), intention to include e-cigarettes/HTPs in home rules was associated with being from Armenia versus Georgia (3.82 vs 2.72, p<0.001), being female versus male (3.91 vs 3.18, p<0.001), having education higher than high school versus lower than high school (3.71 vs 2.97, p=0.003), being married or cohabitating versus other (3.73 vs 3.11, p=0.008), having children in the home versus not having children in the home (3.87 vs 3.21, p=0.003), no past-month smoking versus past-month smoking (4.00 vs 2.90, p<0.001), lower e-cigarette/HTP use intentions (r= -0.101; p=0.019), and higher perceived e-cigarette/HTP risk (r=0.335, p<0.001).

Adjusted multilevel linear regression analysis (Table 2) revealed that factors associated with intention to include e-cigarettes/HTPs in home rules were having children (β=0.42; 95% CI: 0.02–0.82, p=0.042), no past-month smoking (β= -0.82; 95% CI: -1.40 – -0.23, p=0.007), and greater perceived e-cigarette/HTP risk (β=0.38; 95% CI: 0.25–0.50, p<0.001) (Selected findings are presented in Figure 1c.)

## DISCUSSION

Our research contributes valuable insights regarding restrictions on the use of emerging tobacco products like e-cigarettes and HTPs in distinct locations. Specifically, we examined the integration of e-cigarettes and HTPs into smoke-free restrictions within private settings (e.g. homes and cars) in two middle-income countries, Armenia and Georgia, characterized by elevated smoking rates and the recent enactment of nationwide smoke-free policies^18,19^. We found that roughly three-quarters of participants reported having smoke-free home rules (72.9%) and car rules (81.3%), but one to two out of 10 participants chose to exclude e-cigarettes and/or HTPs from their home rules (13.5%) or car rules (18.7%). These results underscore a common theme in the literature – smoke-free restrictions frequently overlook alternative tobacco products^14,30^. While the existing research has primarily focused on these gaps in public policies^14,30^, current findings advance the literature by documenting that this gap extends to private settings such as homes and cars, emphasizing the need for focused assessment and intervention efforts.

Aligning with Social Cognitive Theory^23^ and prior research^31,32^, we found that intentions to use e-cigarettes/HTPs were associated with excluding these products from their home rules, and past-month cigarette use was associated with lower intentions to include e-cigarettes/HTPs in home restrictions. Furthermore, a key cognitive factor – specifically greater perceived e-cigarette/HTP risk – was associated with including e-cigarettes/HTPs in home rules and car rules as well as next-year intentions to include e-cigarettes/HTPs in home rules, which aligns with prior findings^33^.

While perceived social acceptability was not significantly associated with the outcomes, other social influences were significant: having children and no other household smokers was associated with including e-cigarettes/HTPs in car rules, and having children were associated with intent to include e-cigarettes/HTPs in home rules. These findings are consistent with prior research indicating stricter policies in homes with children^34^ and a lower likelihood of including e-cigarettes/HTPs in car rules among those with a family member who smokes^31^. However, having children in the household was not associated with including e-cigarettes/HTPs in smoke-free home rules. One study showed that about one-third of households with children did not ban these products indoors or in cars^30^, underscoring the need to address this gap and capitalize on the higher intentions to include e-cigarettes/HTPs in home policies in this population.

Another factor potentially related to social norms – country of residence – was associated with the likelihood of including e-cigarettes/HTPs in smoke-free home rules. Specifically, participants in Georgia reported greater likelihood compared to those in Armenia. This may be due to earlier enforcement of the national smoke-free policy in Georgia (2018) relative to Armenia (2022), as evidence indicates that comprehensive tobacco control policies promote voluntary smoke-free rules in private settings, such as homes and cars^35,36^. Our findings suggest that comprehensive smoke-free policies in public areas may also lead to including e-cigarettes/HTPs in rules for private settings. Interestingly, the country of residence was not associated with including e-cigarettes/HTPs in car rules or the intention to establish inclusive home rules among those without smoke-free home rules. One plausible explanation is that, despite higher intentions to ban these products among Armenians (as found in bivariate analysis), Georgians who were compelled to adopt such rules may have done so after the law’s implementation in 2018. We also found that those with full versus partial smoke-free rules for private areas were more likely to include e-cigarettes/HTPs in those rules, which is consistent with other research^8^. Notably, e-cigarette and HTP use is more likely in private and public places where bans are in place for smoking but not explicitly for alternative tobacco products^33^, underscoring the importance of intentionally establishing and communicating the inclusion of these products in such rules.

Current findings carry significant implications for research and practice. First, given that private settings may represent one of the most prominent sources of alternative tobacco byproduct exposure, understanding this complex, understudied issue is crucial to identifying opportunities for targeted interventions to raise awareness of the potential harms of these products^32,34^. Second, the evidence base regarding the harms and health consequences associated with the use and byproducts of e-cigarettes/HTPs must be enhanced in order to provide the basis for such interventions. Third, these findings underscore the essential role of comprehensive policies – both in public and private settings. Participants living in a country with a longer standing national smoker-free policy (Georgia) were more likely to include e-cigarettes/HTPs in their home rules, and participants who had established full restrictions in their homes and cars were more likely to include these products in their rules. However, the extent to which these rules are explicit, well-communicated, and well-known by others – and the extent of compliance with these rules – warrants research.

### Limitations

The current study should be interpreted in light of some limitations. First, findings may not generalize to other countries or regions with different cultural, social, and regulatory contexts. Furthermore, findings may not generalize to the general populations of these countries as this study excluded the capital cities and more rural areas; however, the cities included in this study represent approximately one-third of each country’s population. Second, findings may have been impacted by the use of different sampling and recruitment methods across countries due to the available census data. Third, the cross-sectional design precludes inferences regarding causal relationships. Fourth, certain limitations to the data and sample (e.g. self-report measures, unaccounted-for factors, limited power for certain analyses, residual confounding) may have impacted findings.

## CONCLUSIONS

While a significant number of adults in Armenia and Georgia reported having smoke-free home and car rules, one to two out of 10 excluded e-cigarettes and HTPs from these restrictions. Perceived risk was a particularly salient predictor of including them or intending to include them in their rules for personal settings. Key social factors (i.e. other smokers and children in the home) and residing in a country with longer standing public smoke-free restrictions (i.e. Georgia) were also important factors associated with these outcomes. Collectively, findings from this study stress the necessity for a multifaceted approach, combining comprehensive policies, policy reinforcement, targeted education, and community engagement to effectively address the evolving challenge of alternative tobacco product use, particularly within private settings.

## Data Availability

The data supporting this research are available from the authors on reasonable request.
